# ENU Mutagenesis Reveals a Novel Phenotype of Reduced Limb Strength in Mice Lacking Fibrillin 2

**DOI:** 10.1371/journal.pone.0009137

**Published:** 2010-02-09

**Authors:** Gaynor Miller, Monica Neilan, Ruth Chia, Nabeia Gheryani, Natalie Holt, Annabelle Charbit, Sara Wells, Valter Tucci, Zuzanne Lalanne, Paul Denny, Elizabeth M. C. Fisher, Michael Cheeseman, Graham N. Askew, T. Neil Dear

**Affiliations:** 1 Mammalian Genetics of Disease Unit, School of Medicine, University of Sheffield, Sheffield, United Kingdom; 2 Department of Neurodegenerative Disease, UCL Institute of Neurology, London, United Kingdom; 3 Institute of Integrative and Comparative Biology, University of Leeds, Leeds, United Kingdom; 4 Mary Lyon Centre and Mammalian Genetics Unit, Medical Research Council, Harwell, United Kingdom; 5 Leeds Institute of Molecular Medicine, Wellcome Trust Brenner Building, St. James's University Hospital, Leeds, United Kingdom; University Medical Center Groningen, Netherlands

## Abstract

**Background:**

Fibrillins 1 (FBN1) and 2 (FBN2) are components of microfibrils, microfilaments that are present in many connective tissues, either alone or in association with elastin. Marfan's syndrome and congenital contractural arachnodactyly (CCA) result from dominant mutations in the genes *FBN1* and *FBN2* respectively. Patients with both conditions often present with specific muscle atrophy or weakness, yet this has not been reported in the mouse models. In the case of *Fbn1*, this is due to perinatal lethality of the homozygous null mice making measurements of strength difficult. In the case of *Fbn2*, four different mutant alleles have been described in the mouse and in all cases syndactyly was reported as the defining phenotypic feature of homozygotes.

**Methodology/Principal Findings:**

As part of a large-scale N-ethyl-N-nitrosourea (ENU) mutagenesis screen, we identified a mouse mutant, Mariusz, which exhibited muscle weakness along with hindlimb syndactyly. We identified an amber nonsense mutation in *Fbn2* in this mouse mutant. Examination of a previously characterised *Fbn2*-null mutant, *Fbn2^fp^*, identified a similar muscle weakness phenotype. The two *Fbn2* mutant alleles complement each other confirming that the weakness is the result of a lack of Fbn2 activity. Skeletal muscle from mutants proved to be abnormal with higher than average numbers of fibres with centrally placed nuclei, an indicator that there are some regenerating muscle fibres. Physiological tests indicated that the mutant muscle produces significantly less maximal force, possibly as a result of the muscles being relatively smaller in Mariusz mice.

**Conclusions:**

These findings indicate that Fbn2 is involved in integrity of structures required for strength in limb movement. As human patients with mutations in the fibrillin genes *FBN1* and *FBN2* often present with muscle weakness and atrophy as a symptom, *Fbn2*-null mice will be a useful model for examining this aspect of the disease process further.

## Introduction

Inherited neuromuscular disorders have a significant impact on health and cause a wide variety of symptoms such as muscle weakness, cardiomyopathy and/or mental retardation. Their severity also varies significantly from being mild with little or no symptoms (e.g. some cases of myotonic dystrophy) to severe where life expectancy is shortened (e.g. Duchenne muscular dystrophy). The primary tissue affected with each disorder ranges from the nerves connecting the brain and spine to the muscles (e.g. spinal muscular atrophy), the muscles themselves (e.g. Duchenne muscular dystrophy) and the neuromuscular junctions (e.g. myasthenia gravis).

In contrast to neuromuscular disorders connective tissue disorders primarily affect the skin, cardiovascular system, skeleton and eyes. Two such connective tissue disorders are Marfan's syndrome and Congenital Contractural Arachnodactyly (CCA), which are caused by dominant mutations in the fibrillin-1 (*FBN1*) and fibrillin-2 (*FBN2*) genes respectively [1], [2]. Fibrillins are large cysteine-rich glycoproteins found the extracellular matrix of extensive tissues such as the lung, skin and blood vessels. Fibrillins assemble into microfibrils that then become networks providing structural support for the formation of elastic fibres. These scaffolds of microfibrils also act as targets for growth factors controlling differentiation and morphogenesis [3]. Not surprisingly, mutations in the fibrillin proteins can have serious consequences. For example patients with Marfan's syndrome are usually very tall, and can have one or more of the following symptoms: long thin limbs, stretchy skin, hypermobile joints, arachnodactyly, coloboma of the iris, scoliosis, learning disabilities, aneurysms and hypotonia, to name a few [4]. Patients with CCA, on the other hand, whilst having some of the skeletal features in common with Marfan's syndrome, have a crumpled appearance of the ear helix and congenital contractures, and do not typically have the ocular and cardiovascular complications seen with Marfan's syndrome [5]. Of particular relevance to the study described here is that both diseases affect the skeletal muscle seen as hypotonia in Marfan's syndrome and hypoplasia in CCA.

Mouse null mutants have been generated for both the *Fbn1* and *Fbn2* genes and they prove to be imperfect models of the diseases. *Fbn1*-null mutants do display the vascular abnormalities that have been associated with Marfan's syndrome but they do not appear to manifest most other symptoms seen in humans with the condition [6], while reported *Fbn2*-null mutants do not fully recapitulate the CCA phenotype [7]–[9].

ENU mutagenesis is a valuable tool for uncovering mutations that affect muscle development and function. Herein, we report the identification of a mutant, Mariusz, in a recessive ENU mutagenesis screen. Homozygous Mariusz mice suffer from a profound muscle weakness and fusion of hind paw digits. We have identified that Mariusz mice carry a nonsense mutation in the *Fbn2* gene. This mutant is an important model for studying the pathogenic mechanisms underlying muscle weakness and identifies a phenotype, hitherto undiscovered, that appears to result from the absence of Fbn2 protein.

## Results

### The Identification of the Mariusz Mutant

**Figure 1:**
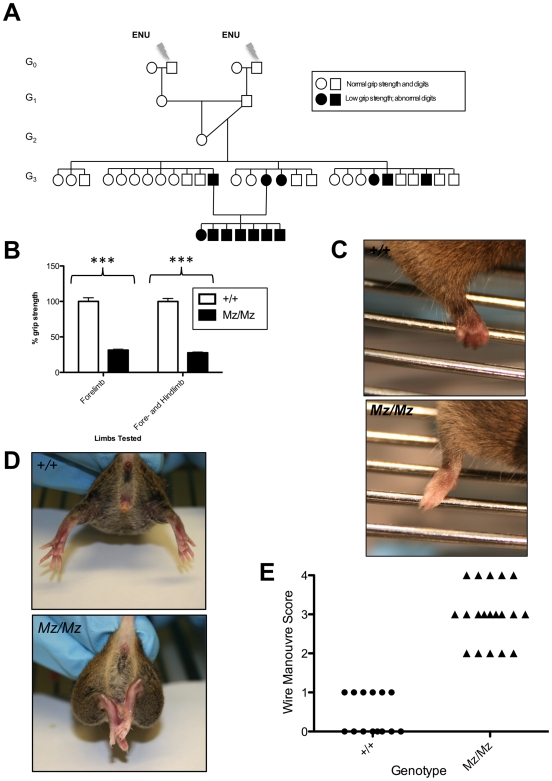
The muscle weakness phenotype of the Mariusz mutant. (**A**) The original pedigree in which the Mariusz mutant was identified. (**B**) Grip-strength comparison using 3-month-old Mariusz homozygotes (n = 30) and wild type (n = 26) littermate controls. The average force generated by wild type mice has been set as 100%. Mean and SD are shown. The differences are highly significant; P-values (Student's t test): *** *P*<0.0001. (**C**) Close-up photos showing the different grasp of Mariusz homozygotes (*Mz/Mz*) and wild type littermate controls. (**D**) Example of clasping phenotype observed in a Mariusz homozygote and littermate control. (**E**) A wire manoeuvre test of Mariusz mice and littermate controls. Scores are as follows: 0 = mouse swings hindlegs on to bar and makes way to edge rapidly; 1 = mouse swings hindlegs on to bar after a delay and makes way to edge rapidly; 2 = mouse is suspended by using underarms to straddle bar and does not lift hind limbs, eventually falls; 3 = mouse falls after short delay; 4 = mouse falls immediately.

We carried out a mutagenesis screen to identify mouse mutants that might possess muscular defects on the basis of exhibiting reduced grip strength. ENU-mutagenized BALB/c males were crossed with wild type C3H/HeH females. A female and male G_1_ offspring of two separate ENU-treated males were intercrossed to maximize the number of mutations being carried in the G_2_ offspring. To generate homozygosity for mutations, G_2_ mice were backcrossed to one of the G_1_ parents to generate G_3_ offspring that were then screened. G_3_ offspring were examined for grip strength in both forelimbs alone, and forelimbs and hindlimbs together. One G_3_ pedigree (Figure 1A) contained mice with very low grip strength (Figure 1B). This phenotype was inherited in an autosomal recessive fashion with complete penetrance in both sexes (Figure 1A). We named this mutant line Mariusz (after the world's strongest man at the time, Mariusz Pudzianowski).

In grip strength tests, the Mariusz mice were unable to grip effectively. The paws of Mariusz mice were not closed but instead remained in an open position with the digits extended (Figure 1C). Although this might account for their poor performance in this test, the overall defect in muscular strength is more profound and involved more than an inability to grip. This was exemplified by suspending the mice by the tail. Whereas wild type mice are able to extend their hindlimbs for a long period of time as they struggle, Mariusz mice could extend their hindlimbs only briefly before they closed against their body, a so-called ‘clasping’ phenotype (Figure 1D). This phenotype does not require any ability to grip. They also performed poorly in the wire manoeuvre test [10] (Figure 1E), another test for muscular strength. Indeed, the loss of strength is so acute that mice could be accurately and simply phenotyped for low muscular strength without any specialized equipment, merely by holding the mice by the tail and assessing their ability to struggle or grip a cage lid.

**Figure 2:**
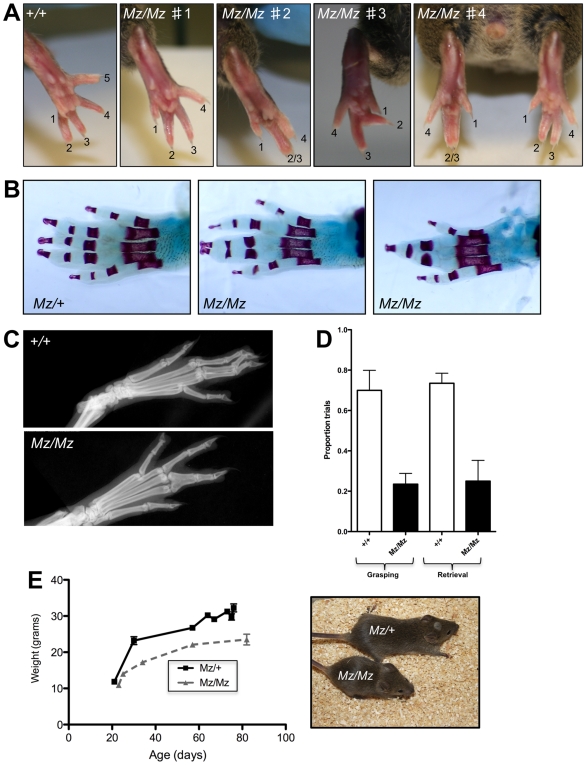
The digit fusion phenotype of the Mariusz mutant. (**A**) Examples of different fusion phenotypes observed - four toes (#1), four toes with two toes fused (#2), four toes with skewed digit (#3), mixture of two digit phenotypes in a single mouse (#4). (**B**) Hard and soft tissue fusion seen in Mariusz mice. Alcian Blue/Alizarin Red-stained left hindlimbs of P7 mice showing a littermate heterozygous control (left), soft tissue syndactyly of toes 3 and 4 (centre), and syndactyly of toes 2,3 and 4 with fusion of the distal phalanges (right). (**C**) X-ray analysis of a hindlimb of a Mariusz mutant demonstrating fusion of the soft tissue as well as the distal phalanx bones. (**D**) Morag test on Mariusz mice and wild type littermates. The proportion of trials in which the mice were able to grasp the food pellets and retrieve the pellets to their mouths are shown. (**E**) Mariusz mice have a reduced body weight. Growth curves of Mariusz male mice and littermate controls. Picture shows a typical example of the size difference between an adult Mariusz female mouse and a littermate control at 3 months of age.

Concomitant with the low grip strength we noted that the mice exhibited hindlimb paw syndactyly of two or more adjacent toes. Both phenotypes - fused digits and weak muscular strength - are fully penetrant in mutants but there is some variability between Mariusz mice for the type of syndactyly. It was most commonly observed as four toes (Figure 2A, *Mz/Mz* #1), though less commonly as four toes with two of the toes fused (Figure 2A, *Mz/Mz* #2), four toes with one of the toes skewed (Figure 2A, *Mz/Mz* #3), or occasionally, even a mixture of two digit phenotypes in the hindlimbs of a single mouse (Figure 2A, *Mz/Mz* #4). The phenotype was the result of a combination of hard and soft tissue fusion. Alcian Blue/Alizarin Red staining and X-rays of hindlimbs showed that digit fusion sometimes resulted from the soft tissue fusing but leaving the phalanges unfused (Figure 2B, Mz/Mz #1) or was the result of fusion of two, or even three, distinct distal phalanx bones and the intervening soft tissue (Figure 2B, Mz/Mz #2; Figure 2C).

The defect in the digits was not responsible for the low grip strength. This is because, firstly, weak grip strength is observed in the forelimbs alone (Figure 1B) although there are no observable digit abnormalities in the forelimb paws. Also, as detailed above, the mice exhibit a startling generalized muscle weakness that did not require the ability to grip. There was 100% concordance for low grip strength and hindlimb digit fusion in abnormal mice from this line and heterozygotes showed no abnormal phenotype (data not shown). This suggested that the two defects might be the result of a single mutation.

Some other phenotypic abnormalities were noted in Mariusz mice. They performed poorly in the MoRAG test for measuring grasping and retrieval of food pellets [11] (Figure 2D). The low score in grasping mimics the results seen in the grip strength test. The poor score in retrieval (where successful retrieval is scored if the mouse brings the food toward the mouth) might indicate a distinct abnormality in motor function. Mariusz mice also exhibited slower growth rates than their littermate controls and were, as a result, smaller (Figure 2E).

### Positional Cloning of the Mariusz Mutation

**Figure 3:**
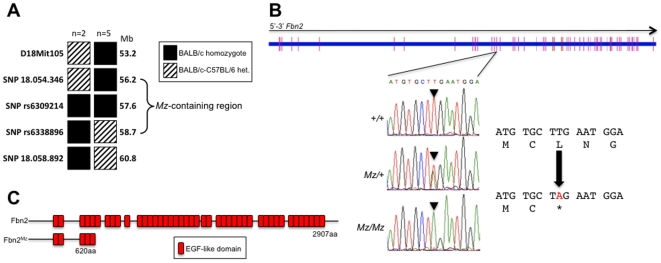
Genetic linkage analysis and positional cloning of the *Mz* mutation. (**A**) Genotypes of seven informative recombinants exhibiting the Mariusz phenotype. The mutation was localized to a 2.5 Mb interval (indicated by the bracketed region). (**B**) Sequence of part of exon 14 of the *Fbn2* gene reveals a T-A substitution in the Mariusz allele. The reading frame of part of exon 14 is shown with the amino acid substitution that results. (**C**) Schematic of the Fbn2 wild type protein and the truncation that results from the Mariusz mutation.

The Mariusz mutation was generated on a BALB/cAnNCrl genetic background, and then crossed to C3H/HeH. A total of 13 G_3_ C3H/HeH x BALB/cAnNCrl mice with the Mariusz phenotype were examined with a panel of 53 markers spanning the entire genome at regular intervals that are polymorphic between these two strains. The strongest linkage signal (LOD score = 3.3) was observed on chromosome 18 at a marker located around 62 Mb. Fine mapping was achieved by backcrossing Mariusz mice to C3H/HeH, C3H/HeN and, finally, to C57BL/6J mice and examining mice with the Mariusz phenotype (low grip strength and fused toes) with a panel of polymorphic markers spanning the region. Genotyping of 310 mice derived from intercrossing Mariusz heterozygotes identified 7 informative recombinants that narrowed the mutation-containing region to a maximum of 2.5 Mb (Figure 3A). At this stage we were unable to find any further polymorphic markers in this region that differentiated between BALB/cAnNCrl and either C3H or C57BL/6J. According to the Ensembl database, eighteen predicted or known genes were located within this region at the time of sequencing. We decided to prioritize candidates for sequencing based on gene structure and known mutant phenotypes; ENU mutants are biased towards mutations in genes with longer coding sequences and more exons [12]. There was one obvious candidate gene in the region–*Fbn2*, with a coding sequence length of 10.5 kb and containing 67 exons. Moreover, 4 mutants of *Fbn2* had previously been characterized, and in all these mutants digit fusion was the defining phenotype reported [7]–[9].

Sequencing of *Fbn2* identified a single mutation in Mariusz mice; a T/A to A/T transversion in exon 14 of *Fbn2* (Figure 3B). The mutation results in the replacement of a codon for leucine (TTG) by a stop codon (TAG) (Figure 3B). This truncates the predicted Fbn2 protein from 2907 to 620 amino acids (Figure 3C). There was complete concordance between animals exhibiting the Mariusz phenotype and possessing the *Fbn2* mutation. We retrospectively regenotyped 145 DNA samples from mice with the Mariusz phenotype and all contained the mutation.

### Examination of Muscle Strength in the *Fbn2^fp^* Mutant

One aspect of our identification of this mutation was confusing. The Mariusz mice clearly had a major defect in muscular strength, yet this had not been reported in any of the other *Fbn2* mutant mice. There were two possible explanations for this discrepancy. Either other *Fbn2* mutants did possess this defect and the phenotype had been missed or the muscle phenotype was not related to the *Fbn2* mutation we identified but was due to a second mutation in a neighbouring gene or intergenic region. The second possibility was highly unlikely as, based on the known ENU-induced mutation rate for this strain and ENU dose (1 mutation per 1.8 Mb) [13], the probability of there being any further mutations anywhere in this region was extremely low (*P* = 0.0001) [14].

**Figure 4:**
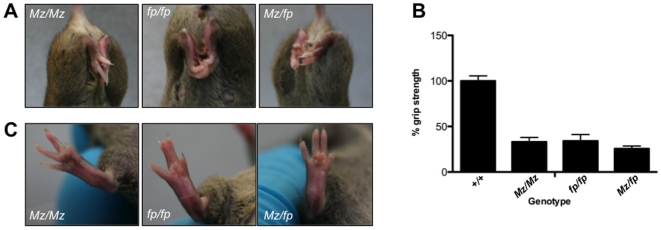
Complementation of the *Fbn2^Mz^* and *Fbn2^fp^* mutations. (**A**) Typical examples of the clasping phenotype of *Fbn2^Mz^*/*Fbn2^Mz^* (*Mz/Mz*) and *Fbn2^fp^/Fbn2^fp^ (fp/fp)* parents and an *Fbn2^Mz^/Fbn2^fp^ (Mz/fp)* offspring. (**B**) Grip strength of mice of the three different genotypes (n = 5 males each group). (**C**) Typical digit phenotypes of the *Mz/Mz* and *fp/fp* parents and a *Mz/fp* offspring.

Nevertheless, to formally exclude the latter possibility we obtained another *Fbn2* mutant (*Fbn2^fp^*). This *Fbn2* mutation is a single nucleotide deletion in exon 39 and results in an absence of Fbn2 protein [8]. It had been previously documented to have syndactyly [8] but there was no report of loss of muscular strength. We observed that *Fbn2^fp^*/*Fbn2^fp^* homozygotes had a profound muscle weakness and were similar to Mariusz mice in both the clasping (Figure 4A) and the grip strength (Figure 4B) tests. Moreover, intercrossing of *Fbn2^fp^*/*Fbn2^fp^* x *Fbn2^Mz^*/*Fbn2^Mz^* resulted in complementation. All *Fbn2^fp^*/*Fbn2^Mz^* offspring had profound muscle weakness and syndactyly (Figures 4A–C) similar to both parent phenotypes indicating that the two mutations were recessive alleles of the same gene. This is unequivocal evidence that null mutations in *Fbn2* result in a loss of muscular strength.

### Physiological Assessment of Muscle in *Fbn2*-Null Mice

**Figure 5:**
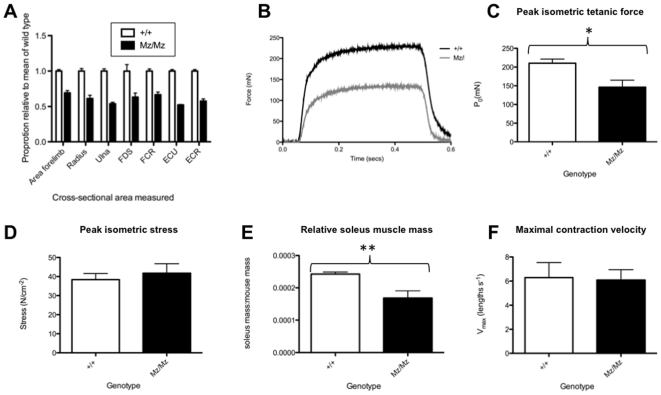
Muscle force measurements in Mariusz mice. (**A**) Morphometric measurement of cross-sectional area of Mariusz muscles in histological sections normalized to the area of the muscle in wild type controls (n = 3 mice each genotype at 5 weeks of age; n = 50 fibres counted per sample). Muscle abbreviations are: FDS, Flexor Digitorum Sublimis; FCR, Flexor Carpi Radialis; ECU, Extensor Carpi Ulnaris; ECR, Extensor Carpi Radialis. ‘Area forelimb’ refers to the entire area of the forelimb to emphasize the size difference between Mariusz mice and littermates controls. (**B**) Example trace of a tetanic contraction in a Mariusz soleus muscle and a wild type control. (**C**) Mean peak tetanic force for control (n = 5) and Mariusz (n = 6) mice with SEM shown by error bars. (**D**) Mean isometric stress of the soleus muscle for wild type (n = 5) and Mariusz (n = 6) mice with SEM shown by error bars. (**E**) The mean ratio of the mass of the soleus muscle to the mass of the mouse for control (n = 5) and Mariusz (n = 6) mice with SEM shown by error bars. (**F**) The mean maximum contraction velocity of the soleus muscle in wild type control (n = 4) and Mariusz (n = 5) mice with SEM shown by error bars. P-values: * *P*<0.05; ** *P*<0.01.

The loss of muscular strength could be due to manifestations of the mutation in the tendons, skeletal muscle, peripheral nervous system or central nervous system, all of which express Fbn2 [15], [16]. Tendons of *Fbn2*-null mice have been previously analyzed and no structural alterations were reported, though the level of collagen cross-linking was reduced [17]. Examination of histological sections of 5-week-old Mariusz mice and littermate controls showed a general reduction in cross-sectional area of the muscles of Mariusz mice (Figure 5A), which might account for at least part of the reduction in muscular strength.

Detailed measurements of soleus muscle function were undertaken in 4-month-old Mariusz mice and wild type controls. The peak isometric tetanic force (P_0_) produced by the soleus muscle was significantly higher in the controls compared to the Mariusz mice (*P*<0.05) (Figure 5B,C). However, the peak isometric stress was not significantly different between the control and Mariusz mice (Figure 5D). This would suggest that the myofibrillar structure of the muscle is unaffected by the absence of Fbn2 protein. The difference in force between muscle from Mariusz mice and controls in these tests could have been accounted for by the difference in the mass of the soleus muscle (6.72±0.17 mg (mean ± SEM) in Mariusz mice compared with 3.93±0.47 mg in controls; *P*<0.01), and hence physiological cross-sectional area. In part this is due to the Mariusz mice being significantly lighter than the controls (Mariusz: 23.7±0.70 g (mean ± SEM) compared to control: 27.7±0.41 g (*P*<0.05)). However, Mariusz mice also have relatively smaller soleus muscles than the controls (Figure 5E; *P*<0.01). The maximal contraction velocity (V_max_) was not significantly different from that of the controls (*P*>0.05) (Figure 5F) and the twitch time (time to peak twitch) was not significantly different between the two groups (*P*>0.05).

### Mariusz Mice Exhibit Centronuclear Muscle Fibres

**Figure 6:**
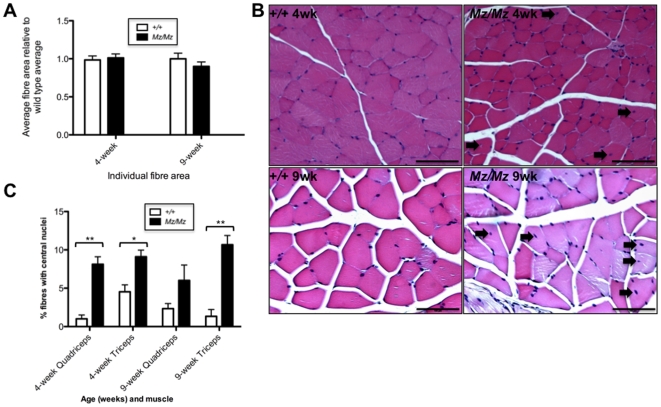
Skeletal muscle histology in Mariusz mice. (**A**) Morphometric analysis of muscle fibre cross-sectional area in the soleus and quadriceps muscles in Mariusz (n = 3) and wild type controls (n = 3). Two-way ANOVA was not significant. (**B**) Typical H&E sections of Mariusz quadriceps muscle from mice at 4 and 9 weeks of age. Scale bar = 100 um. (**C**) Quantification of central nucleation. The number of muscle fibres with central nuclei is represented as percentage of the total number of muscle fibres analyzed in >100 fibres. Arrows indicate centrally displaced nuclei. Mice were aged 4 weeks and 9 weeks (n = 3 *Mz/Mz*; n = 3*+/+*). P-values (Student's t test): * *P*<0.05, ** *P*<0.01.

We examined the skeletal muscle of Mariusz mutants. Morphometric analysis of fibre size showed no significant difference between Mariusz and control mice (Figure 6A). However, an abnormally high number of fibres with centrally placed nuclei were evident in all muscles examined from all mice (Figure 6B, C). This occurred consistently in all muscle samples from all mice we examined. Such a finding is indicative of regenerating myofibres and is considered myopathic. It is a feature of some muscle diseases and suggests that some fibres have degenerated at some point. There was no evidence of inflammation.

## Discussion

Using ENU mutagenesis, we have identified a *Fbn2* recessive mutation that results in muscle weakness, in addition to the classic syndactyly phenotype reported in similar *Fbn2* mutants [7]–[9]. *FBN2* mutations are responsible for many cases of CCA [2] while mutation of the related gene *FBN1* can result in Marfan's syndrome [1]. Marfan's syndrome and CCA are somewhat indistinct from a clinical perspective and muscular abnormalities have been reported in both diseases. In Marfan's syndrome, myopathy, as reflected in one or more of poor muscle development, lack of response to exercise and hypotonia, is not uncommon [18]. It has been suggested it may be present in the majority of cases [19]. Muscular abnormalities have been reported in CCA patients. Muscle hypoplasia, particularly of the calf muscles, is a clinical feature of CCA [5] while atrophy has been reported in CCA [20], or syndromes with some features of CCA [21].

Gene targeting in embryonic stem cells has been used to generate null alleles of *Fbn1* and *Fbn2* and both have proven to be partial but imperfect models of Marfan's syndrome and CCA respectively. *Fbn1*-null mice have a similar vascular phenotype to that of Marfan's patients [6] while *Fbn2*-null mice exhibit the transient postnatal contractures as seen in CCA [7].

Five other recessive mouse mutants have been described for *Fbn2*. A null allele generated by gene targeting in embryonic stem cells (*Fbn2^tm1Rmz^*) [7], a chemically mutagenized ES cell mutant allele *Fbn2^fp-3J^* [9], two spontaneous mutant alleles *Fbn2^fp^* and *Fbn2^fp-2J^* [8], and the *sy* mutant, which has a complete *Fbn2* deletion [8], [22]. Bilateral syndactyly is the main reported phenotype in all. It is likely that these five alleles, along with the *Fbn2^Mz^* allele, result in reduced, or complete loss of, Fbn2 function. Both the *Fbn2^fp^* and *Fbn2^fp-2J^* mutants have deletions causing frameshifts that result in truncation of the Fbn2 protein [8] while the *Fbn2^fp-3J^* allele is due to a LINE insertion that also truncates the protein [9]. In none of these cases has there be any report of weakness in the limbs, despite the fact that our testing showed that the *Fbn2^fp^* homozygotes previously described as exhibiting only syndactyly [8] also suffer from muscular weakness identical to Mariusz mice. Although we cannot confirm that the *Fbn2^tm1Rmz^*, *Fbn2^fp-2J^* and *Fbn2^fp-3J^* alleles also result in a homozygous phenotype of reduced strength, we feel it is highly likely given the likely effects of these mutant alleles on the Fbn2 protein and the similar digit fusion phenotype. As Fbn1 along with Fbn2, contributes to myofibrils, it would be worthwhile generating mice with a conditional null *Fbn1* allele so that their muscular phenotype could be examined.

Physiological tests on the muscle indicate that the myofibre structure is not altered in Fbn2-null mice (as their was no difference in peak isometric stress). The muscle itself generated less force, probably due its relatively smaller size in *Fbn2*-null mice. However, the loss of muscular strength in *Fbn2*-null mice is more extreme than the reduction in the maximum force produced by the muscle itself, suggesting an extra-muscular involvement. Our finding that the muscle itself shows signs of degeneration/regeneration suggests that some of the myofibres of *Fbn2*-null mice are in a regenerative phase. However, there was no gross inflammation and the level of regeneration can probably not account for the drastic reduction in the strength of these mice. A reduced level of collagen cross-linking has been identified in the tendons of *Fbn2*-null mice [17]. As this provides structural integrity to the tendons, the mechanical properties of the tendon, such as the maximal tensile strength, might be altered as a result. However, a reduced maximum tensile strength would make them more susceptible to rupture, and there is no evidence of this.

We propose that there is a significant alteration in a structure or structures external to the muscle that account for some of the loss of strength. One possibility is that it lies within the nervous system. In preliminary investigations, dorsal horn motor neuron counts appeared normal (data not shown). Given that many structures could potentially be affected, further analysis for any neuropathology would be worthwhile.

Why has this muscle weakness phenotype been missed in the original description of the *Fbn2^fp^*/*Fbn2^fp^* homozygotes? In a blind test of several experienced animal technicians at three separate institutes we provided with the Mariusz mice, although all were able to identify the syndactyly phenotype quickly, none identified the grip strength/clasping phenotype by mere handling alone. Once they were made aware of the phenotype they were easily able to identify mutant *Fbn2*-null mice with reduced strength based on either testing the ability of the mouse to struggle when held by the tail or testing the ability of the mouse to grip the wire grating on a standard cage lid. Thus the phenotype, although easy to discern without specialized equipment, required a rigorous phenotyping assay to enable the initial identification.

Our report herein emphasizes an additional value of ENU mutagenesis screens. Even if the mutation is of a known gene for which a similarly acting mutation has already been identified, it can still uncover hitherto unidentified phenotypes associated with mutant alleles. It further emphasizes the value of extensive phenotyping tests on mouse mutants to uncover new mutant phenotypes [23]. Such an aim has been a driving force behind the large-scale standardized phenotyping programmmes such as the European Mouse Disease Clinic [24], the German Mouse Clinic [25] and the Wellcome Trust Sanger Centre Mouse Genetics Programme. The discovery of a new phenotype in *Fbn2* mutants emphasizes how such screens will aid in determining gene function in the post genomic era.

### Conclusion

We have identified a new phenotype - loss of strength - resulting from absence of Fbn2. The loss of strength is at least partly the result of relatively smaller muscles in *Fbn2*-null mice, but extra-muscular effects account for a significant proportion of the strength loss. Given that muscular weakness and muscular atrophy are common findings in patients suffering from Marfan's syndrome and CCA, *Fbn2*-null mice may be a good model for investigating this aspect of the disease further.

## Materials and Methods

### Mice

Mice were maintained in high health status facilities according to the UK Home Office guidelines with access to food and water *ad libidum*. *Fbn2^fp^* mice were obtained from the Jackson Laboratories. All animal studies were approved both the ethical committees of the Medical Research Council, Harwell, University of Sheffield and University of Leeds as well as by the UK Home Office.

### ENU Mutagenesis

Adult BALB/cAnNCrl mice aged 10 weeks were mutagenized by intraperitoneal injection of two weekly doses of 100 mg/kg ENU and after recovery of fertility were mated with C3H/HeH. F_1_ offspring were intercrossed and the G_2_ offspring were backcrossed to G_1_ parents to generate the G_3_ generation for screening.

### Phenotyping Tests

Mice were assessed for their performance in the grip strength test using a mouse grip strength meter (Bioseb, Vitrolles Cedex, France). Mice were held by the tail and removed from their cage. They were gently lowered onto the grid and allowed to place either all four paws, or their forelimb paws only, on the mesh. The mice were then gently pulled back steadily and slowly across the grid while recording the maximum grip strength in Newtons. For each animal 4 measurements were taken to obtain an average value for grip strength for each mouse. For the wire manoeuvre test, the method and scoring system of Rafael *et al*. [10] was used. The MORAG test was performed and scored as previously described [11].

### Genetic Mapping

Genome-wide low-resolution mapping was performed using DNA samples from 13 G_3_ hybrids. Genomic DNA samples isolated from tail biopsies of these animals were screened by PCR amplification and gel electrophoresis with 53 microsatellite markers spaced at regular intervals across the genome. For finer mapping, the Mariusz mutation was sequentially backcrossed and intercrossed on both C3H/HeN and C57BL/6J backgrounds. Microsatellite markers polymorphic between either BALB/c and C3H or BALB/c and C57BL/6J were used to identify Mariusz mice that were recombinant in the critical region. Single nucleotide polymorphisms (SNPs) were genotyped by sequencing of PCR products amplified with primers flanking the SNP site. Sequencing of candidate genes involved designing primer pairs to amplify individual exons as well as flanking splice donor/acceptor sequences. For DNA sequencing, all exons and splice sites of selected genes were sequenced using homozygous Mariusz DNA and compared to wild type DNA from all strains.

### Genotyping of Cohorts for Experimental Analysis

For experimental analysis of mutant and wild type cohorts, mice were deemed to be carrying the mutation if they either contained BALB/c-derived markers for the region containing the Mariusz mutation on chromosome 16 or later, after the mutation was identified, by direct sequencing of PCR products to identify if they carried the T-A mutation in *Fbn2*.

### H&E of Skeletal Muscle

The quadriceps and triceps muscle from Mariusz and C57BL/6J age-matched mice were dissected and formalin fixed before being embedded in paraffin wax. Transverse sections were taken and stained with haematoxylin and eosin.

### Muscle Physiology

Mice were sacrificed by cervical dislocation. The soleus muscle was immediately dissected out from the hindlimb in oxygenated Krebs-Henseleit Ringers solution at 3°C [composition in mmol l^−1^: NaCl, 118.5; NaHCO_3_, 25.0; KCl, 4.8; MgSO_4_, 1.2; KH_2_PO_4_, 1.2; CaCl_2_, 1.4; glucose, 11.0; saturated with 95% O_2_, 5%CO_2_ [26]. The proximal end of the muscle was attached to the base of a Perspex™ muscle chamber using a stainless steel spring clip to clamp the proximal tendon. The muscle was attached to the arm of a muscle lever (model 300B-LR, Aurora Scientific, Aurora, ON, Canada) *via* a lightweight silver chain attached to the distal tendon of the soleus muscle using 5-0 silk suture. The soleus muscle was held vertically within the muscle chamber that was circulated with oxygenated Krebs-Henseleit Ringer's solution. The temperature of the Ringer's solution was gradually increased from 3°C to 37°C over a period of approximately 15 minutes. The muscle was left for at least 30 minutes following the dissection before the contractile properties were measured.

A series of isometric twitches were used to set the length of the muscle to that at which maximum twitch force was produced (*L*_0_). Supramaximal stimuli were delivered *via* parallel platinum electrodes running alongside the muscle (pulse width 0.25 ms). The motor arm of the muscle lever was mounted on an adjustable stand that enabled the length of the muscle to be varied. An isometric tetanic contraction was produced using a train of stimuli delivered at the fusion frequency of the muscle (typically 150 Hz). Muscle force was recorded at 5 kHz *via* an A/D board (DAS1802AO, Keithley Instruments, Theale, UK) and the maximal isometric tetanic force produced by the muscle (*P*_0_) calculated_._ Force-velocity characteristics were determined during after-loaded isotonic tetanic contractions. Muscle length was set to 1.05 *L*_0_ so that the muscle shortened through the plateau of the length-force relationship. The muscle was maximally stimulated; force was allowed to rise to a predefined level (ranging from approximately 5 to 80% of *P*_0_) and was then held constant by muscle shortening. Further isometric tetanic contractions were performed periodically during and at the end of the series of isotonic contractions as references of maximum isometric force in order to monitor and correct for any decline in muscle performance. Any decline was assumed to be linear between controls. Muscle force (*P*) and length were recorded at 5 kHz *via* an A/D board (DAS1802AO, Keithley Instruments, Theale, UK). Muscle length (*L*) was converted to strain [(*L*−*L*_0_)/*L*_0_]. Strain was differentiated with respect to time in order to determine velocity. This was plotted against corrected relative force (*P*/*P*_0_). A hyperbolic-linear relationship was fit to these data and the maximum velocity of shortening (*V*_max_) estimated by extrapolation to zero force [27].

Following the measurements of the contractile properties of the muscle, the muscle was blotted and weighed. The physiological cross-sectional area of the muscle was calculated by dividing the muscle mass by mean fibre length (0.85 *L*_0_) [28] and dividing by muscle density (1060 kg m^−3^) [29]. Maximum muscle stress was calculated as *P*_0_ divided by physiological cross-sectional area.

### Software and Databases

DNA sequence alignments for examining sequence data were performed using DNASTAR (DNASTAR Inc. Madison, WI). Prism software (GraphPad Software, Inc., La Jolla, CA) was used for statistical analysis. Morphometric analysis of muscle fibre cross-sectional area was estimated using Image J [30]. For meaurement of fibre cross-sectional area, contrast of micrographs was enhanced by 0.5%. The freehand tool was then used to mark individual fibres.
